# 2-Azido-*N*-(4-methyl­phen­yl)acetamide

**DOI:** 10.1107/S2414314622006216

**Published:** 2022-07-29

**Authors:** Mohcine Missioui, Walid Guerrab, Abdulsalam Alsubari, Joel T. Mague, Youssef Ramli

**Affiliations:** a Laboratory of Medicinal Chemistry, Drug Sciences Research Center, Faculty of Medicine and Pharmacy, Mohammed V University in Rabat, Morocco; b Laboratory of Medicinal Chemistry, Faculty of Clinical Pharmacy, 21 September University, Yemen; c Department of Chemistry, Tulane University, New Orleans, LA 70118, USA; Sunway University, Malaysia

**Keywords:** crystal structure, acetamide, azide, hydrogen bond

## Abstract

The asymmetric unit of the title compound, C_9_H_10_N_4_O, comprises three independent mol­ecules, two pairs of which differ significantly in the rotational orientation of the azido group and one pair having very similar conformations; the N—N—C—C torsion angles are −173.9 (2), −102.7 (2) and −173.6 (2)°. In the crystal, each independent mol­ecule forms N—H⋯O hydrogen bonds with its glide-plane-related counterparts, forming zigzag chains extending along the *c*-axis direction.

## Abstract 2

The asymmetric unit comprises three independent mol­ecules, two pairs of which differ significantly in the rotational orientation of the azido group and one pair having very similar conformations. Zigzag chains extending along the *c*-axis direction are formed individually by each independent mol­ecule through N—H⋯O hydrogen bonds.

## Graphical abstract

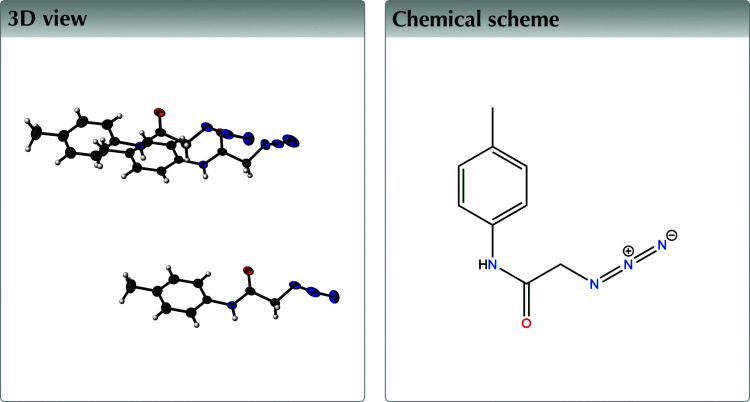

## Structure description

*N*-aryl­acetamides are significant inter­mediates for the synthesis of medicinal, agrochemical and pharmaceutical compounds (Beccalli *et al.*, 2007 ▸; Valeur & Bradley, 2009 ▸; Allen & Williams, 2011 ▸; Missioui *et al.*, 2021 ▸, 2022*a* ▸,*b* ▸). Azides have found valuable applications in medicinal chemistry (Contin *et al.*, 2019 ▸), mol­ecular biology (Ahmed & Abdallah, 2019 ▸) and attract increasing attention in the field of organic synthesis as inter­mediates for the preparation of heterocycles such as tetra­zoles, triazolines, triazoles, *etc* (Chauhan *et al.*, 2019 ▸; Bakulev *et al.*, 2019 ▸; Abad *et al.*, 2020 ▸; Missioui, Lgaz *et al.*, 2022 ▸). Based on the aforementioned information and in continuation of our research efforts to synthesize more *N*-aryl­acetamides (Missioui *et al.*, 2020 ▸; Missioui, Guerrab, Nchioua *et al.*, 2022 ▸; Guerrab *et al.*, 2021 ▸), we report the synthesis and crystal structure of the title compound. The structure of the closely related compound 2-azido-*N*-(4-fluorophenyl)acetamide is reported by Missioui, Guerrab, Alsubari *et al.* (2022 ▸).

**Figure 1:**
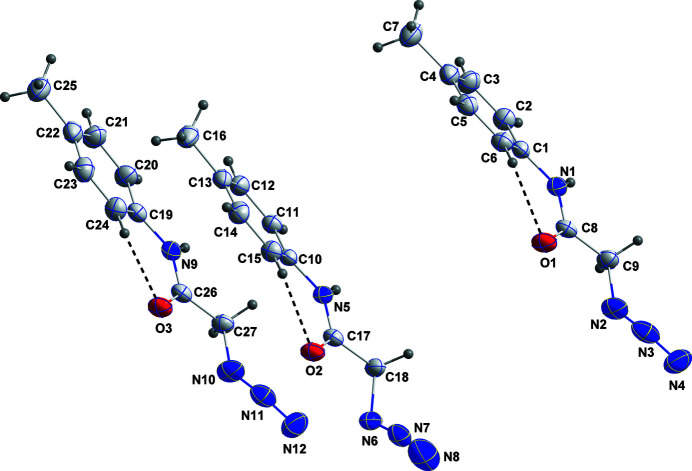
The asymmetric unit with the labelling scheme and 50% probability ellipsoids. The intra­molecular C—H⋯O hydrogen bonds are shown as dashed lines.

The asymmetric unit comprises three independent mol­ecules with the azide moieties oriented in opposite directions between mol­ecules containing O1 and O2 but with the same situation in the mol­ecules containing O2 and O3 (Table 1 ▸). On the other hand, the mol­ecules containing O1 and O3 have very similar conformations. The rotational orientations of the phenyl groups with respect to the carboxamide moieties are partially determined by intra­molecular C—H⋯O hydrogen bonds (Fig. 1 ▸ and Table 2 ▸).

**Figure 2:**
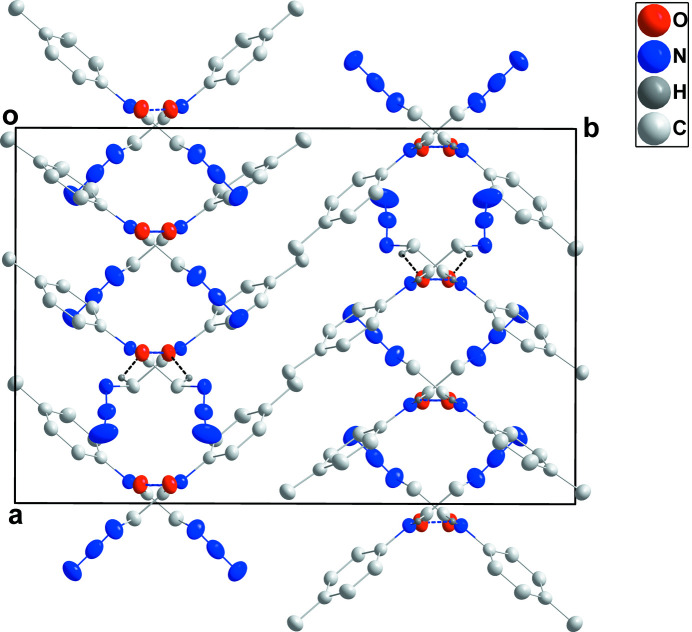
Packing viewed along the *c*-axis direction with N—H⋯O and C—H⋯O hydrogen bonds indicated, respectively, by blue and black dashed lines.

In the crystal, each component of the asymmetric unit forms a chain with its counterparts related by the glide plane and extending along the *c*-axis direction through N1—H1*A*⋯O1 or N5—H5*A*⋯O2 or N9—H9*C*⋯O3 hydrogen bonds (Table 2 ▸). In the case of the mol­ecule containing O2, the chain is reinforced by C18—H18*B*⋯O2 hydrogen bonds (Table 2 ▸ and Figs. 2 ▸ and 3 ▸). The chains pack through normal van der Waals contacts.

## Synthesis and crystallization

2-Chloro-*N*-(*p*-tol­yl)acetamide (0.011 mol) and sodium azide (0.015 mol) were dissolved in a mixture of ethanol/water (70:30) then refluxed for 24 h at 80°C. After completion of the reaction (monitored by thin-layer chromatography, TLC), the 2-azido-*N*-(4-methyl­phen­yl)acetamide precipitate was filtered and washed with cold water. A portion of the product was dissolved in hot ethanol, the solution was filtered and the filtrate was left undisturbed for 7 days to form colourless plate-like crystals.

Yield 73%, mp 360–362 K, FT–IR (ATR, ν, cm^−1^) 3254 ν(N—H amide), 3073 ν(C—H_arom_), 2961 ν(C—H,CH_2_), 2109 ν (N_3_), 1027 ν(N—C amide), 1660 ν(C=O amide), 1175 υ(C—N). ^1^H NMR (DMSO–*d*_6_) δ p.p.m. 4.02 (2*H*, *s*, CH_2_), 4.21 (3*H*, *s*, CH_3_), 6.93–7.1 (4*H*, *m*, *J* = 1.3 Hz, H_arom_), 10.05 (1*H*, *s*, NH), ^13^C NMR (DMSO–*d*_6_) δ p.p.m. 51.18 (CH_2_), 63.85 (CH_3_), 131.47 (C_arom_—N), 155.47 (C_arom-_–O), 113.90–120.86 (C_arom_); 165.71 (C=O); HRMS (ESI MS) (*m*/*z*) calculated for C_9_H_10_N_4_O 190.21 found 190.1191.

## Refinement

Crystal data, data collection and structure and refinement details are summarized in Table 3 ▸.

## Supplementary Material

Crystal structure: contains datablock(s) global, I. DOI: 10.1107/S2414314622006216/tk4079sup1.cif

Structure factors: contains datablock(s) I. DOI: 10.1107/S2414314622006216/tk4079Isup2.hkl

Supporting information file. DOI: 10.1107/S2414314622006216/tk4079Isup3.cml

CCDC reference: 2178858

Additional supporting information:  crystallographic information; 3D view; checkCIF report

## Figures and Tables

**Figure 3:**
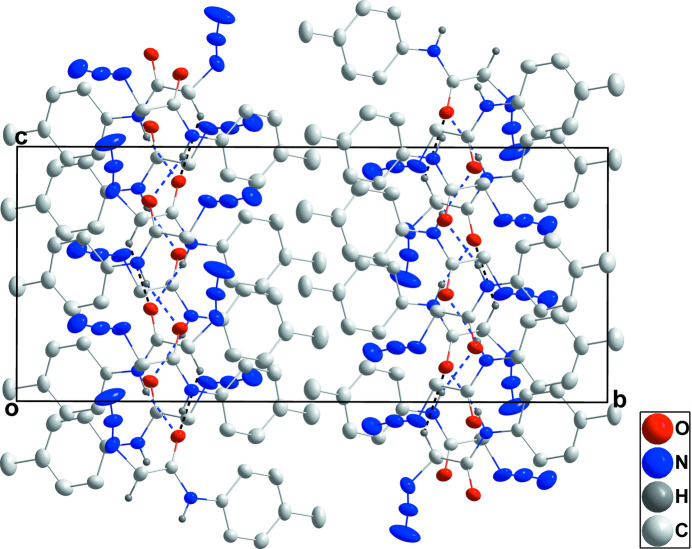
Packing viewed along the *a*-axis direction with N—H⋯O and C—H⋯O hydrogen bonds indicated, respectively, by blue and black dashed lines.

**Table 1:** Selected torsion angles (°)

N3—N2—C9—C8	−173.9 (2)	N11—N10—C27—C26	−173.6 (2)
N7—N6—C18—C17	−102.7 (2)		

**Table 2:** Hydrogen-bond geometry (Å, °)

*D*—H⋯*A*	*D*—H	H⋯*A*	*D*⋯*A*	*D*—H⋯*A*
N1—H1*A*⋯O1^i^	0.86 (3)	2.04 (3)	2.867 (2)	162 (2)
N5—H5*A*⋯O2^i^	0.85 (3)	2.01 (3)	2.833 (2)	163 (2)
C15—H15⋯O2	0.91 (3)	2.34 (2)	2.905 (3)	120 (2)
C18—H18*B*⋯O2^i^	0.99 (3)	2.57 (3)	3.353 (3)	136 (2)
N9—H9*C*⋯O3^i^	0.81 (3)	2.05 (3)	2.835 (2)	163 (2)
C24—H24⋯O3	0.91 (3)	2.27 (3)	2.882 (3)	125 (2)

Symmetry code: (i) 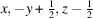 .

**Table 3:** Experimental details

Crystal data	
Chemical formula	C_9_H_10_N_4_O
*M*_r_	190.21
Crystal system, space group	Monoclinic, *P*2_1_/*c*
Temperature (K)	150
*a*, *b*, *c* (Å)	14.4362 (4), 21.3403 (6), 9.2949 (3)
β (°)	98.356 (1)
*V* (Å^3^)	2833.11 (14)
*Z*	12
Radiation type	Cu *K*α
μ (mm^−1^)	0.77
Crystal size (mm)	0.22 × 0.16 × 0.08

Data collection	
Diffractometer	Bruker D8 VENTURE PHOTON 100 CMOS
Absorption correction	Multi-scan (*SADABS*; Krause *et al.*, 2015 ▸)
*T*_min_, *T*_max_	0.86, 0.94
No. of measured, independent and observed [*I* > 2σ(*I*)] reflections	21650, 5486, 4161
*R*_int_	0.039
(sin θ/λ)_max_ (Å^−1^)	0.617

Refinement	
*R*[*F* ^2^ > 2σ(*F* ^2^)], *wR*(*F* ^2^), *S*	0.059, 0.178, 1.05
No. of reflections	5486
No. of parameters	466
H-atom treatment	H atoms treated by a mixture of independent and constrained refinement
Δρ_max_, Δρ_min_ (e Å^−3^)	0.44, −0.39

Computer programs: *APEX3* and *SAINT* (Bruker, 2016 ▸), *SHELXT* (Sheldrick, 2015*a* ▸), *SHELXL 2018/1* (Sheldrick, 2015*b* ▸), *DIAMOND* (Brandenburg & Putz, 2012 ▸) and *SHELXTL* (Sheldrick, 2008 ▸).

## full crystallographic data

### Crystal data

**Table:**

C_9_H_10_N_4_O	*F*(000) = 1200
*M**_r_* = 190.21	*D*_x_ = 1.338 Mg m^−^^3^
Monoclinic, *P*2_1_/*c*	Cu *K*α radiation, λ = 1.54178 Å
*a* = 14.4362 (4) Å	Cell parameters from 9919 reflections
*b* = 21.3403 (6) Å	θ = 3.7–72.2°
*c* = 9.2949 (3) Å	µ = 0.77 mm^−^^1^
β = 98.356 (1)°	*T* = 150 K
*V* = 2833.11 (14) Å^3^	Plate, colourless
*Z* = 12	0.22 × 0.16 × 0.08 mm

### Data collection

**Table:**

Bruker D8 VENTURE PHOTON 100 CMOS diffractometer	5486 independent reflections
Radiation source: INCOATEC IµS micro–focus source	4161 reflections with *I* > 2σ(*I*)
Mirror monochromator	*R*_int_ = 0.039
Detector resolution: 10.4167 pixels mm^-1^	θ_max_ = 72.2°, θ_min_ = 3.7°
ω scans	*h* = −17→17
Absorption correction: multi-scan (*SADABS*; Krause *et al.*, 2015)	*k* = −26→24
*T*_min_ = 0.86, *T*_max_ = 0.94	*l* = −10→11
21650 measured reflections	

### Refinement

**Table:**

Refinement on *F*^2^	Primary atom site location: structure-invariant direct methods
Least-squares matrix: full	Secondary atom site location: difference Fourier map
*R*[*F*^2^ > 2σ(*F*^2^)] = 0.059	Hydrogen site location: mixed
*wR*(*F*^2^) = 0.178	H atoms treated by a mixture of independent and constrained refinement
*S* = 1.05	*w* = 1/[σ^2^(*F*_o_^2^) + (0.0988*P*)^2^ + 1.2801*P*] where *P* = (*F*_o_^2^ + 2*F*_c_^2^)/3
5486 reflections	(Δ/σ)_max_ = 0.002
466 parameters	Δρ_max_ = 0.44 e Å^−^^3^
0 restraints	Δρ_min_ = −0.39 e Å^−^^3^

### Special details

**Table:**

Geometry. All esds (except the esd in the dihedral angle between two l.s. planes) are estimated using the full covariance matrix. The cell esds are taken into account individually in the estimation of esds in distances, angles and torsion angles; correlations between esds in cell parameters are only used when they are defined by crystal symmetry. An approximate (isotropic) treatment of cell esds is used for estimating esds involving l.s. planes.
Refinement. Refinement of F^2^ against ALL reflections. The weighted R-factor wR and goodness of fit S are based on F^2^, conventional R-factors R are based on F, with F set to zero for negative F^2^. The threshold expression of F^2^ > 2sigma(F^2^) is used only for calculating R-factors(gt) etc. and is not relevant to the choice of reflections for refinement. R-factors based on F^2^ are statistically about twice as large as those based on F, and R- factors based on ALL data will be even larger. The methyl group hydrogen atoms were included as riding contributions in idealized positions since independent refinement yielded unsatisfactory geometries.The H atoms were treated by a mixture of independent and constrained refinement.

### Fractional atomic coordinates and isotropic or equivalent isotropic displacement parameters (Å^2^)

**Table:**

	*x*	*y*	*z*	*U*_iso_*/*U*_eq_	
O1	0.94983 (12)	0.27610 (7)	0.28617 (15)	0.0374 (4)	
N1	0.94218 (12)	0.29612 (8)	0.04434 (18)	0.0288 (4)	
H1A	0.9534 (17)	0.2808 (11)	−0.037 (3)	0.036 (6)*	
N2	1.06934 (16)	0.17717 (11)	0.2860 (2)	0.0456 (5)	
N3	1.12261 (17)	0.14178 (11)	0.2867 (2)	0.0462 (5)	
N4	1.18070 (17)	0.10247 (12)	0.3057 (3)	0.0584 (6)	
C1	0.87815 (13)	0.34695 (9)	0.0273 (2)	0.0271 (4)	
C2	0.82911 (16)	0.35703 (10)	−0.1109 (2)	0.0342 (5)	
H2	0.8403 (17)	0.3316 (11)	−0.190 (3)	0.036 (6)*	
C3	0.76610 (16)	0.40609 (11)	−0.1351 (3)	0.0395 (5)	
H3	0.7357 (18)	0.4126 (12)	−0.230 (3)	0.044 (7)*	
C4	0.75000 (15)	0.44650 (10)	−0.0236 (3)	0.0361 (5)	
C5	0.80085 (16)	0.43586 (10)	0.1134 (3)	0.0358 (5)	
H5	0.7923 (17)	0.4632 (12)	0.195 (3)	0.040 (7)*	
C6	0.86475 (16)	0.38713 (10)	0.1402 (2)	0.0324 (5)	
H6	0.8968 (18)	0.3818 (12)	0.240 (3)	0.051 (8)*	
C7	0.68108 (17)	0.49939 (12)	−0.0490 (3)	0.0491 (6)	
H7A	0.684547	0.518373	−0.144064	0.074*	
H7B	0.695979	0.531009	0.027316	0.074*	
H7C	0.617706	0.483320	−0.046823	0.074*	
C8	0.97286 (14)	0.26489 (9)	0.1665 (2)	0.0278 (4)	
C9	1.04041 (16)	0.21228 (10)	0.1437 (2)	0.0328 (5)	
H9A	1.094 (2)	0.2312 (13)	0.113 (3)	0.051 (8)*	
H9B	1.0123 (18)	0.1829 (13)	0.072 (3)	0.046 (7)*	
O2	0.59911 (11)	0.27335 (7)	0.86318 (15)	0.0342 (4)	
N5	0.58708 (12)	0.29502 (8)	0.62103 (18)	0.0267 (4)	
H5A	0.5974 (17)	0.2809 (11)	0.539 (3)	0.036 (6)*	
N6	0.68901 (13)	0.16221 (9)	0.8282 (2)	0.0382 (4)	
N7	0.75608 (14)	0.16342 (10)	0.9230 (2)	0.0404 (5)	
N8	0.81422 (19)	0.15780 (17)	1.0172 (3)	0.0832 (10)	
C10	0.52692 (14)	0.34800 (9)	0.6098 (2)	0.0256 (4)	
C11	0.47922 (14)	0.36246 (9)	0.4726 (2)	0.0284 (4)	
H11	0.4846 (15)	0.3348 (10)	0.395 (3)	0.029 (6)*	
C12	0.42233 (15)	0.41482 (10)	0.4533 (2)	0.0333 (5)	
H12	0.3878 (18)	0.4254 (12)	0.350 (3)	0.044 (7)*	
C13	0.41091 (14)	0.45438 (9)	0.5689 (2)	0.0330 (5)	
C14	0.45925 (16)	0.43892 (10)	0.7051 (3)	0.0362 (5)	
H14	0.451 (2)	0.4667 (14)	0.782 (3)	0.063 (9)*	
C15	0.51679 (15)	0.38677 (10)	0.7267 (2)	0.0320 (5)	
H15	0.5482 (16)	0.3763 (11)	0.815 (3)	0.036 (6)*	
C16	0.35033 (16)	0.51175 (11)	0.5467 (3)	0.0451 (6)	
H16A	0.371814	0.538444	0.472383	0.068*	
H16B	0.354393	0.534995	0.638346	0.068*	
H16C	0.285253	0.499254	0.514934	0.068*	
C17	0.61908 (14)	0.26242 (9)	0.7411 (2)	0.0262 (4)	
C18	0.68726 (16)	0.21025 (10)	0.7153 (2)	0.0318 (5)	
H18A	0.7514 (19)	0.2291 (12)	0.716 (3)	0.043 (7)*	
H18B	0.666 (2)	0.1898 (13)	0.621 (3)	0.056 (8)*	
O3	0.27488 (11)	0.27395 (7)	0.57857 (15)	0.0359 (4)	
N9	0.26399 (12)	0.29490 (8)	0.33643 (18)	0.0276 (4)	
H9C	0.2770 (17)	0.2798 (11)	0.262 (3)	0.034 (6)*	
N10	0.39415 (17)	0.17693 (11)	0.5742 (2)	0.0497 (6)	
N11	0.45011 (15)	0.13998 (10)	0.5754 (2)	0.0418 (5)	
N12	0.50716 (17)	0.10135 (11)	0.5955 (2)	0.0545 (6)	
C19	0.20449 (14)	0.34791 (9)	0.3227 (2)	0.0272 (4)	
C20	0.16642 (16)	0.36623 (11)	0.1823 (2)	0.0370 (5)	
H20	0.1734 (19)	0.3414 (13)	0.104 (3)	0.049 (7)*	
C21	0.11114 (17)	0.41922 (12)	0.1608 (3)	0.0430 (5)	
H21	0.0838 (19)	0.4294 (13)	0.063 (3)	0.053 (8)*	
C22	0.09127 (15)	0.45572 (10)	0.2763 (3)	0.0381 (5)	
C23	0.12924 (16)	0.43601 (11)	0.4155 (3)	0.0395 (5)	
H23	0.118 (2)	0.4638 (13)	0.502 (3)	0.055 (8)*	
C24	0.18568 (16)	0.38350 (11)	0.4397 (2)	0.0351 (5)	
H24	0.2128 (19)	0.3704 (13)	0.529 (3)	0.050 (8)*	
C25	0.03161 (18)	0.51349 (12)	0.2526 (3)	0.0531 (7)	
H25A	0.044391	0.534972	0.164345	0.080*	
H25B	0.046129	0.541573	0.336221	0.080*	
H25C	−0.034625	0.501632	0.241599	0.080*	
C26	0.29622 (14)	0.26290 (9)	0.4575 (2)	0.0270 (4)	
C27	0.36337 (16)	0.21023 (10)	0.4325 (2)	0.0316 (5)	
H27A	0.4158 (18)	0.2284 (11)	0.399 (3)	0.037 (6)*	
H27B	0.333 (2)	0.1813 (13)	0.366 (3)	0.053 (8)*	

### Atomic displacement parameters (Å^2^)

**Table:**

	*U*^11^	*U*^22^	*U*^33^	*U*^12^	*U*^13^	*U*^23^
O1	0.0544 (10)	0.0360 (8)	0.0244 (7)	0.0067 (7)	0.0146 (7)	0.0036 (6)
N1	0.0348 (9)	0.0308 (9)	0.0221 (8)	0.0018 (7)	0.0092 (7)	−0.0002 (6)
N2	0.0641 (14)	0.0517 (12)	0.0258 (10)	0.0102 (11)	0.0228 (9)	0.0092 (8)
N3	0.0592 (14)	0.0516 (13)	0.0277 (10)	−0.0166 (12)	0.0066 (9)	0.0056 (9)
N4	0.0638 (15)	0.0573 (14)	0.0541 (14)	0.0200 (12)	0.0080 (11)	0.0149 (11)
C1	0.0274 (9)	0.0265 (10)	0.0287 (10)	−0.0027 (8)	0.0083 (8)	0.0028 (7)
C2	0.0373 (11)	0.0365 (11)	0.0288 (11)	−0.0003 (9)	0.0050 (9)	−0.0025 (9)
C3	0.0377 (12)	0.0432 (13)	0.0359 (13)	0.0038 (10)	−0.0009 (10)	0.0036 (9)
C4	0.0290 (10)	0.0327 (11)	0.0466 (13)	−0.0024 (9)	0.0058 (9)	0.0012 (9)
C5	0.0375 (11)	0.0316 (11)	0.0397 (12)	−0.0028 (9)	0.0107 (9)	−0.0030 (9)
C6	0.0383 (11)	0.0300 (10)	0.0298 (11)	−0.0016 (9)	0.0078 (9)	−0.0001 (8)
C7	0.0408 (13)	0.0408 (13)	0.0640 (17)	0.0075 (11)	0.0014 (12)	0.0005 (11)
C8	0.0334 (10)	0.0275 (9)	0.0230 (10)	−0.0058 (8)	0.0056 (8)	0.0012 (7)
C9	0.0382 (12)	0.0355 (11)	0.0256 (10)	0.0047 (9)	0.0074 (9)	0.0036 (8)
O2	0.0474 (9)	0.0323 (8)	0.0246 (7)	0.0015 (6)	0.0107 (6)	0.0040 (6)
N5	0.0342 (9)	0.0254 (8)	0.0218 (8)	0.0029 (7)	0.0089 (7)	0.0001 (6)
N6	0.0411 (10)	0.0329 (10)	0.0402 (10)	0.0004 (8)	0.0046 (9)	0.0099 (8)
N7	0.0440 (11)	0.0531 (12)	0.0256 (10)	−0.0055 (9)	0.0095 (9)	0.0069 (8)
N8	0.0636 (16)	0.136 (3)	0.0447 (15)	−0.0191 (17)	−0.0087 (13)	0.0369 (16)
C10	0.0290 (9)	0.0224 (9)	0.0272 (10)	−0.0019 (7)	0.0097 (8)	0.0010 (7)
C11	0.0316 (10)	0.0279 (10)	0.0261 (10)	−0.0016 (8)	0.0057 (8)	−0.0003 (8)
C12	0.0299 (10)	0.0327 (11)	0.0372 (12)	−0.0009 (9)	0.0039 (9)	0.0051 (9)
C13	0.0290 (10)	0.0246 (10)	0.0468 (12)	−0.0013 (8)	0.0105 (9)	0.0008 (8)
C14	0.0387 (12)	0.0312 (11)	0.0405 (12)	0.0005 (9)	0.0118 (10)	−0.0071 (9)
C15	0.0370 (11)	0.0303 (11)	0.0298 (11)	0.0004 (9)	0.0085 (9)	−0.0029 (8)
C16	0.0371 (12)	0.0292 (11)	0.0695 (17)	0.0033 (10)	0.0090 (11)	−0.0001 (11)
C17	0.0314 (10)	0.0237 (9)	0.0248 (9)	−0.0043 (8)	0.0085 (8)	0.0003 (7)
C18	0.0357 (11)	0.0294 (10)	0.0320 (11)	0.0052 (9)	0.0100 (9)	0.0060 (8)
O3	0.0493 (9)	0.0376 (8)	0.0231 (7)	0.0027 (7)	0.0130 (6)	0.0032 (6)
N9	0.0322 (9)	0.0312 (9)	0.0208 (8)	−0.0001 (7)	0.0083 (7)	−0.0015 (7)
N10	0.0677 (14)	0.0583 (13)	0.0273 (10)	0.0166 (12)	0.0217 (10)	0.0144 (9)
N11	0.0510 (12)	0.0495 (12)	0.0256 (9)	−0.0109 (11)	0.0077 (8)	0.0030 (8)
N12	0.0575 (14)	0.0592 (14)	0.0462 (13)	0.0170 (12)	0.0062 (10)	0.0083 (10)
C19	0.0267 (9)	0.0277 (10)	0.0280 (10)	−0.0053 (8)	0.0065 (8)	0.0006 (7)
C20	0.0426 (12)	0.0409 (12)	0.0285 (11)	0.0046 (10)	0.0081 (9)	0.0007 (9)
C21	0.0431 (13)	0.0475 (14)	0.0381 (13)	0.0075 (11)	0.0045 (10)	0.0076 (10)
C22	0.0284 (10)	0.0334 (11)	0.0531 (14)	−0.0032 (9)	0.0080 (10)	0.0006 (10)
C23	0.0355 (11)	0.0385 (12)	0.0456 (13)	−0.0005 (10)	0.0092 (10)	−0.0086 (10)
C24	0.0375 (11)	0.0390 (12)	0.0292 (11)	0.0012 (9)	0.0060 (9)	−0.0048 (9)
C25	0.0414 (13)	0.0407 (14)	0.077 (2)	0.0072 (11)	0.0082 (13)	0.0038 (12)
C26	0.0305 (10)	0.0269 (9)	0.0245 (9)	−0.0066 (8)	0.0066 (8)	0.0006 (7)
C27	0.0383 (11)	0.0311 (11)	0.0260 (10)	0.0001 (9)	0.0071 (9)	0.0022 (8)

### Geometric parameters (Å, º)

**Table:**

O1—C8	1.230 (2)	C12—H12	1.04 (3)
N1—C8	1.335 (3)	C13—C14	1.393 (3)
N1—C1	1.419 (3)	C13—C16	1.501 (3)
N1—H1A	0.86 (3)	C14—C15	1.386 (3)
N2—N3	1.077 (3)	C14—H14	0.95 (3)
N2—C9	1.525 (3)	C15—H15	0.91 (3)
N3—N4	1.181 (3)	C16—H16A	0.9800
C1—C6	1.390 (3)	C16—H16B	0.9800
C1—C2	1.390 (3)	C16—H16C	0.9800
C2—C3	1.384 (3)	C17—C18	1.528 (3)
C2—H2	0.95 (2)	C18—H18A	1.01 (3)
C3—C4	1.394 (3)	C18—H18B	0.99 (3)
C3—H3	0.94 (3)	O3—C26	1.232 (2)
C4—C5	1.393 (3)	N9—C26	1.341 (3)
C4—C7	1.500 (3)	N9—C19	1.415 (3)
C5—C6	1.388 (3)	N9—H9C	0.81 (3)
C5—H5	0.98 (3)	N10—N11	1.128 (3)
C6—H6	0.98 (3)	N10—C27	1.505 (3)
C7—H7A	0.9800	N11—N12	1.161 (3)
C7—H7B	0.9800	C19—C24	1.386 (3)
C7—H7C	0.9800	C19—C20	1.396 (3)
C8—C9	1.522 (3)	C20—C21	1.382 (3)
C9—H9A	0.96 (3)	C20—H20	0.92 (3)
C9—H9B	0.96 (3)	C21—C22	1.390 (3)
O2—C17	1.233 (2)	C21—H21	0.96 (3)
N5—C17	1.339 (3)	C22—C23	1.394 (4)
N5—C10	1.420 (2)	C22—C25	1.502 (3)
N5—H5A	0.85 (3)	C23—C24	1.384 (3)
N6—N7	1.211 (3)	C23—H23	1.03 (3)
N6—C18	1.465 (3)	C24—H24	0.91 (3)
N7—N8	1.128 (3)	C25—H25A	0.9800
C10—C15	1.390 (3)	C25—H25B	0.9800
C10—C11	1.393 (3)	C25—H25C	0.9800
C11—C12	1.383 (3)	C26—C27	1.524 (3)
C11—H11	0.95 (2)	C27—H27A	0.94 (3)
C12—C13	1.395 (3)	C27—H27B	0.93 (3)
			
C8—N1—C1	127.34 (17)	C13—C14—H14	115.7 (19)
C8—N1—H1A	118.5 (16)	C14—C15—C10	119.6 (2)
C1—N1—H1A	113.2 (16)	C14—C15—H15	122.6 (16)
N3—N2—C9	117.05 (19)	C10—C15—H15	117.7 (16)
N2—N3—N4	171.7 (3)	C13—C16—H16A	109.5
C6—C1—C2	119.58 (19)	C13—C16—H16B	109.5
C6—C1—N1	123.19 (18)	H16A—C16—H16B	109.5
C2—C1—N1	117.21 (18)	C13—C16—H16C	109.5
C3—C2—C1	120.1 (2)	H16A—C16—H16C	109.5
C3—C2—H2	119.1 (15)	H16B—C16—H16C	109.5
C1—C2—H2	120.7 (15)	O2—C17—N5	124.77 (18)
C2—C3—C4	121.6 (2)	O2—C17—C18	121.65 (18)
C2—C3—H3	118.1 (16)	N5—C17—C18	113.54 (16)
C4—C3—H3	120.3 (16)	N6—C18—C17	109.98 (17)
C5—C4—C3	117.2 (2)	N6—C18—H18A	110.7 (15)
C5—C4—C7	121.0 (2)	C17—C18—H18A	108.6 (15)
C3—C4—C7	121.8 (2)	N6—C18—H18B	107.3 (17)
C6—C5—C4	122.3 (2)	C17—C18—H18B	109.6 (16)
C6—C5—H5	117.6 (15)	H18A—C18—H18B	111 (2)
C4—C5—H5	120.1 (15)	C26—N9—C19	127.96 (17)
C5—C6—C1	119.3 (2)	C26—N9—H9C	114.8 (17)
C5—C6—H6	117.8 (16)	C19—N9—H9C	117.0 (17)
C1—C6—H6	122.9 (16)	N11—N10—C27	117.32 (19)
C4—C7—H7A	109.5	N10—N11—N12	171.3 (2)
C4—C7—H7B	109.5	C24—C19—C20	119.0 (2)
H7A—C7—H7B	109.5	C24—C19—N9	123.58 (19)
C4—C7—H7C	109.5	C20—C19—N9	117.38 (18)
H7A—C7—H7C	109.5	C21—C20—C19	120.4 (2)
H7B—C7—H7C	109.5	C21—C20—H20	118.9 (17)
O1—C8—N1	124.90 (19)	C19—C20—H20	120.5 (17)
O1—C8—C9	122.14 (18)	C20—C21—C22	121.8 (2)
N1—C8—C9	112.96 (17)	C20—C21—H21	118.0 (17)
C8—C9—N2	110.01 (17)	C22—C21—H21	120.2 (17)
C8—C9—H9A	107.2 (17)	C21—C22—C23	116.8 (2)
N2—C9—H9A	109.4 (16)	C21—C22—C25	121.7 (2)
C8—C9—H9B	111.3 (16)	C23—C22—C25	121.6 (2)
N2—C9—H9B	108.4 (16)	C24—C23—C22	122.5 (2)
H9A—C9—H9B	110 (2)	C24—C23—H23	119.6 (16)
C17—N5—C10	127.42 (17)	C22—C23—H23	117.8 (16)
C17—N5—H5A	118.5 (16)	C23—C24—C19	119.6 (2)
C10—N5—H5A	113.6 (16)	C23—C24—H24	124.4 (18)
N7—N6—C18	115.80 (19)	C19—C24—H24	116.0 (18)
N8—N7—N6	171.4 (3)	C22—C25—H25A	109.5
C15—C10—C11	119.29 (19)	C22—C25—H25B	109.5
C15—C10—N5	123.33 (18)	H25A—C25—H25B	109.5
C11—C10—N5	117.33 (17)	C22—C25—H25C	109.5
C12—C11—C10	120.22 (19)	H25A—C25—H25C	109.5
C12—C11—H11	121.2 (14)	H25B—C25—H25C	109.5
C10—C11—H11	118.5 (14)	O3—C26—N9	124.54 (19)
C11—C12—C13	121.6 (2)	O3—C26—C27	121.98 (18)
C11—C12—H12	119.5 (14)	N9—C26—C27	113.48 (17)
C13—C12—H12	118.9 (14)	N10—C27—C26	109.16 (17)
C14—C13—C12	117.18 (19)	N10—C27—H27A	109.5 (15)
C14—C13—C16	121.5 (2)	C26—C27—H27A	107.8 (15)
C12—C13—C16	121.3 (2)	N10—C27—H27B	108.3 (18)
C15—C14—C13	122.1 (2)	C26—C27—H27B	109.8 (17)
C15—C14—H14	122.1 (19)	H27A—C27—H27B	112 (2)
			
C8—N1—C1—C6	25.6 (3)	C13—C14—C15—C10	−0.4 (3)
C8—N1—C1—C2	−156.2 (2)	C11—C10—C15—C14	0.3 (3)
C6—C1—C2—C3	−1.2 (3)	N5—C10—C15—C14	177.55 (19)
N1—C1—C2—C3	−179.52 (19)	C10—N5—C17—O2	1.3 (3)
C1—C2—C3—C4	0.1 (3)	C10—N5—C17—C18	−176.63 (18)
C2—C3—C4—C5	0.7 (3)	N7—N6—C18—C17	−102.7 (2)
C2—C3—C4—C7	−179.4 (2)	O2—C17—C18—N6	25.1 (3)
C3—C4—C5—C6	−0.4 (3)	N5—C17—C18—N6	−156.85 (18)
C7—C4—C5—C6	179.7 (2)	C26—N9—C19—C24	14.4 (3)
C4—C5—C6—C1	−0.7 (3)	C26—N9—C19—C20	−168.4 (2)
C2—C1—C6—C5	1.5 (3)	C24—C19—C20—C21	0.2 (3)
N1—C1—C6—C5	179.71 (19)	N9—C19—C20—C21	−177.1 (2)
C1—N1—C8—O1	−0.4 (3)	C19—C20—C21—C22	−0.2 (4)
C1—N1—C8—C9	179.19 (18)	C20—C21—C22—C23	−0.5 (4)
O1—C8—C9—N2	1.9 (3)	C20—C21—C22—C25	179.6 (2)
N1—C8—C9—N2	−177.75 (18)	C21—C22—C23—C24	1.3 (3)
N3—N2—C9—C8	−173.9 (2)	C25—C22—C23—C24	−178.9 (2)
C17—N5—C10—C15	22.5 (3)	C22—C23—C24—C19	−1.3 (4)
C17—N5—C10—C11	−160.22 (19)	C20—C19—C24—C23	0.5 (3)
C15—C10—C11—C12	−0.1 (3)	N9—C19—C24—C23	177.64 (19)
N5—C10—C11—C12	−177.53 (18)	C19—N9—C26—O3	3.1 (3)
C10—C11—C12—C13	0.0 (3)	C19—N9—C26—C27	−176.86 (18)
C11—C12—C13—C14	−0.1 (3)	N11—N10—C27—C26	−173.6 (2)
C11—C12—C13—C16	178.9 (2)	O3—C26—C27—N10	0.8 (3)
C12—C13—C14—C15	0.2 (3)	N9—C26—C27—N10	−179.23 (18)
C16—C13—C14—C15	−178.8 (2)		

### Hydrogen-bond geometry (Å, º)

**Table:**

*D*—H···*A*	*D*—H	H···*A*	*D*···*A*	*D*—H···*A*
N1—H1*A*···O1^i^	0.86 (3)	2.04 (3)	2.867 (2)	162 (2)
N5—H5*A*···O2^i^	0.85 (3)	2.01 (3)	2.833 (2)	163 (2)
C15—H15···O2	0.91 (3)	2.34 (2)	2.905 (3)	120 (2)
C18—H18*B*···O2^i^	0.99 (3)	2.57 (3)	3.353 (3)	136 (2)
N9—H9*C*···O3^i^	0.81 (3)	2.05 (3)	2.835 (2)	163 (2)
C24—H24···O3	0.91 (3)	2.27 (3)	2.882 (3)	125 (2)

Symmetry code: (i) *x*, −*y*+1/2, *z*−1/2.
